# One‐stage random effects meta‐analysis using linear mixed models for aggregate continuous outcome data

**DOI:** 10.1002/jrsm.1331

**Published:** 2019-01-08

**Authors:** Katerina Papadimitropoulou, Theo Stijnen, Olaf M. Dekkers, Saskia le Cessie

**Affiliations:** ^1^ Clinical Epidemiology Leiden University Medical Center Leiden Netherlands; ^2^ Biomedical Data Sciences Leiden University Medical Center Leiden Netherlands

**Keywords:** linear mixed models, meta‐analysis, pseudo individual participant data, random effects model, simulation study

## Abstract

The vast majority of meta‐analyses uses summary/aggregate data retrieved from published studies in contrast to meta‐analysis of individual participant data (IPD). When the outcome is continuous and IPD are available, linear mixed modelling methods can be employed in a one‐stage approach. This allows for flexible modelling of within‐study variability and between‐study effects and accounts for the uncertainty in the estimates of between‐study and within‐study residual variances. However, IPD are seldom available. For the normal outcome case, we present a method to generate pseudo IPD from aggregate data using group mean, standard deviation, and sample sizes within each study, ie, the sufficient statistics. Analyzing the pseudo IPD with likelihood‐based methods yields identical results as the analysis of the unknown true IPD. The advantage of this method is that we can employ the mixed modelling framework, implemented in many statistical software packages, and explore modelling options suitable for IPD, such as fixed study‐specific intercepts and fixed treatment effect model, fixed study‐specific intercepts and random treatment effects, and both random study and treatment effects and different options to model the within‐study residual variance. This allows choosing the most realistic (or potentially complex) residual variance structures across studies, instead of using an overly simple structure. We demonstrate these methods in two empirical datasets in Alzheimer disease, where an extensive model, assuming all within‐study variances to be free, fitted considerably better. In simulations, the pseudo IPD approach showed adequate coverage probability, because it accounted for small sample effects.

## 1 INTRODUCTION

Meta‐analysis is the most common evidence synthesis method to estimate a combined treatment or exposure effect using the individual study findings. Most often these meta‐analyses use group‐level summary statistics (aggregate data; AD) retrieved from published articles and reports, in contrast to meta‐analysis of individual participant/patient data (IPD). In this paper, we focus on meta‐analyses where only aggregate data are available.

The meta‐analysis of aggregate data is traditionally based on two‐stage methods. Here, in the first stage, each study produces an estimate of the effect size and its corresponding standard error. For instance, the estimated effect size may be the (log) odds ratio in case of binary outcomes. In the continuous outcome case, the estimated effect size commonly is the difference between the two group means, with the standard error calculated as the square root of the sum of the squared standard errors of the means. At the second stage, the estimated effect sizes and standard errors form the data input of a standard fixed or random effects meta‐analysis model.^1^, ^2^ Several methods to perform a random effects meta‐analysis have been proposed over the years,^3^ although the method of moments introduced by DerSimonian and Laird (DL)^4^ has prevailed as the most routinely used given its simplicity of computations. Competing heterogeneity estimators include the Paule and Mantel,^5^ Hartung and Macambi,^6^ Hartung and Knapp,^7^ Sidik and Jonkman,^8^ and the restricted maximum likelihood approach (REML).^9^ In this work, we focus on comparing the most widely used methods in practice, the DL and REML approaches including the Hartung‐Knapp correction.^7^

The underlying assumptions of the aforementioned approaches are that each estimated effect size follows a normal distribution with mean equal to the true effect size of that study and variance equal to the squared standard error, where these within‐study variances are assumed to be fixed and known. The true effect sizes then are assumed to follow a normal distribution with mean (called the overall effect size) and variance (called the between‐study variance) to be estimated from the data.

Disadvantages of these two‐stage methods are as follows: (1) the within‐study variances are treated as known, while in fact they are estimated; (2) for continuous outcomes, the assumptions about the variances in the treatment and control group within each study often go unnoticed; (3) for binary outcomes, the within‐study normal distribution assumption might be violated, for instance, in case, the event is rare; and (4) bias could be introduced in the overall effect size by possible correlation between the estimated effect size and its standard error. Technically speaking, these problems are due to the fact that an approximate within‐study likelihood is used instead of the exact likelihood.

When individual patient data are available, one‐stage methods may overcome these difficulties by using the exact distribution of the within‐study data.^10^ For dichotomous outcomes, individual patient data can be extracted in a straightforward manner from the aggregate data when the number of events and sample size are available for both groups.^11^ Next, straightforward random effects logistic regression can be applied using a generalized linear mixed model program, available nowadays in all statistical packages.^12^, ^13^, ^14^, ^15^, ^16^ The advantage of this one‐stage approach is that it avoids the aforementioned problems of the two‐stage approach. Moreover standard statistical software can be used, instead of a special purpose meta‐analysis program, such as RevMan.^17^ Also, for ordinal outcomes (based on the number of responses within each treatment category)^18^ and survival data (reconstructing the data based on Kaplan‐Meier survival curves),^19^ it is possible to reconstruct the individual patient data. For event/person‐years data, also, one‐stage methods have been proposed.^13^

IPD and AD meta‐analyses will provide very similar results, when they are based on the same underlying assumptions, such as common residual variances for control and treatment groups.^20^ However, standard AD methods will yield too small standard errors when the number of studies in the meta‐analyses is small and cannot handle different (potentially complex) residual variance structures across studies. If IPD data are available, this can be easily accounted for using linear mixed modelling. Although it may technically be possible incorporating more complex variance structures in an AD meta analysis, it is not implemented in standard software. Also corrections for a small number of studies, such as the Hartung‐Knapp^7^ correction, are thus far implemented in R and Stata yet not in SAS.

For a meta‐analysis of aggregate continuous outcome data, only two‐stage modelling is used thus far. In this case, the aforementioned disadvantages (3) and (4) of the two‐stage approach do not apply; however, the first one, not accounting for estimation of the within‐study variances, remains and flexibility in modelling the within‐study variances is lacking. For example, it is important to study whether the variances in treatment and control group are equal. In a study comparing treatment with placebo, the variance of the response in the treatment group may be larger than in the control group, in which case separate estimates for the two variances are needed.

In this paper, we propose a one‐stage meta‐analysis approach for the normal outcome case where the mean, standard deviation, and sample size per group are available as summary data. In order to do so, we develop an algorithm to reconstruct IPD, hereinafter referred as pseudo IPD. Since the summary data are the sufficient statistics, the pseudo IPD will have the same likelihood as the unknown original individual data. In this way, we are able to use the flexibility of the linear mixed modelling framework and account for the uncertainty in the within‐study variances without the need of a specialized meta‐analytic software. In this work, we focus on reconstructing IPD, based on sufficient statistics, yet we do not simulate covariates at individual level.

The paper is organized as follows. In Section 2, we introduce two illustrating datasets in Alzheimer disease, where group‐level summary data are available describing plasma levels of micronutrients as the continuous outcomes of interest. In Section 3, we describe the existing modelling options for IPD following an increasing complexity approach, assuming fixed and random effects for study and treatment/exposure effects and different options for modelling the within‐study variance. In Section 4, we introduce our novel approach of constructing pseudo IPD from the aggregate continuous data. In Section 5, we apply the proposed method to the plasma levels datasets and compare the results with standard two‐stage methods on the aggregate data. In Section 6, we conduct a simulation study to examine the performance of our proposed method in comparison with two‐stage methods. Brief final comments are provided in Section 7 with some discussion.

## 2 ILLUSTRATING EXAMPLE

Lopes da Silva et al^21^ performed a systematic review to compare the plasma levels of micronutrients and fatty acids in Alzheimer disease patients with those of elderly controls with normal cognition. The authors identified five or more studies for various plasma levels of interest and performed a random effects meta‐analysis using the REML^9^ method for estimating the between‐study variance, *τ* ^2^. To illustrate our method, we selected two continuous outcomes of interest: the iron blood level measured in five studies of in total 753 participants and the folate level reported in 31 studies of in total 4555 patients. The mean iron and folate level for each group (control and Alzheimer disease) in each trial are shown in Tables 1 and 2, respectively, with higher values indicating a healthier nutritional profile. For each study, the group‐level summary statistics are shown for the control and the disease group along with the sample size per group.

**Table 1:** Summary data on iron blood levels (μg/dL)

Study name	Control group			Alzheimer disease group		
	Mean	sd	n	Mean	sd	n
Basun 1991	114	25	26	100	39	20
Kristensen 1993	89	32	20	90	33	26
Modashi 1996	63	30	421	56	22	31
Molina 1998	101	31	28	114	35	26
Vural 2010	81	31	50	67	23	50

Abbreviations: μg, microgram; dL, deciliter; n, number of subjects; sd, standard deviation.

**Table 2:** Summary data on folate levels (nmol/L)

Study name	Control group			Alzheimer disease group		
	Mean	sd	n	Mean	sd	n
Agarwal 2010	15.68	30.13	127	14.97	14.74	32
Anello 2004	15.7	5.9	181	14.3	5.7	180
AsitaDeSilva 2005	19.71	9.74	21	15.86	8.38	23
Cascalheira 2009	20.39	1.7	36	18.8	5.3	19
Clarke 1998	22.9	10	108	17.60	10.7	164
Dominguez 2005	29.57	8.97	19	17.87	7.18	29
Faux 2011	30.29	12.68	760	29.35	14.46	205
Galimberti 2008	19.82	6.16	23	8.63	2.81	29
Galluci 2004	14.05	11.1	42	11.55	6.12	137
Hogervorst 2002	24.92	11.33	62	15.86	11.33	66
Irizarry 2005	35.20	32.9	88	29.9	21.3	145
Joosten 1997	8.61	3.2	49	7.93	4.2	52
Karimi 2009	15.86	8.61	49	14.5	6.57	51
Koseoglu 2007	28.09	3.4	40	21.41	4.40	51
Lelhuber 2000	14.27	9.281	19	9.97	3.4	19
Li 2004	37.20	21.2	30	29.2	12.7	30
Linnebank 2010	14.05	7.74	60	15.62	7.04	60
Lovati 2007	15.56	7.93	76	8.19	5.32	108
Malaguarnera 2004	13.6	3.18	30	10.6	3.16	30
Mizrahi 2004	4.8	2.6	155	4.3	3.2	75
Morillas‐Ruiz 2010	28.8	7.71	48	21.81	8.71	52
Parnetti 1992	14.05	1.12	26	9.46	1.07	52
Postiglione 2001	8.5	3.2	74	5.7	2.1	74
Quadri 2005	16.8	5.5	79	13.1	5.9	111
Ravaglia 2000	11.5	1.2	13	8	0.5	34
Ravaglia 2004	16.57	7.26	29	11.1	4.3	51
Regland 1992	20	18	32	16.7	15.46	53
Religa 2003	17.13	12.21	100	19.28	7.66	99
Selley 2002	25.09	4.7	25	14.74	4.26	27
Serot 2001	13.16	4.83	28	12.12	4.87	30
Villa 2009	19.03	4.08	18	16.77	4.69	20

Abbreviations: L, liter; n, number of subjects; nmol, nanomole; sd, standard deviation.

We performed a two‐step random effects meta‐analysis using the package *metafor* 22 in R 3.4.1^23^ on the two datasets and estimated the pooled effect sizes and the between‐study variance by the standard DerSimonian and Laird^4^ (DL), the REML^9^ method, and the REML approach with the Hartung‐Knapp correction,^7^ which aims to adjust for the small number of studies. The iron blood level analysis showed a mean difference between the groups equal to −5.57 μg/dL (−14.16 to 3.02) for the DL method, −5.52 μg/L (−14.28 to 3.24) using the REML approach, and −5.52 μg/L (−18.57 to 7.53) using the Hartung‐Knapp correction.^7^ The estimated between‐study variances were similar: 43.9 (DL) vs 47.3 (REML). The analysis on the folate dataset showed significantly lower plasma levels of folate in the Alzheimer disease patients compared with the healthy controls and −3.88 nmol/L (−5.13 to −2.64) for the REML method while the mean difference between the groups was equal to −3.80 nmol/L with a more narrow confidence interval of (−4.76 to −2.84) when using the DL method. The between‐study variance estimated under the DL method was smaller than the REML approach: 5.17 vs 9.88.

## 3 LINEAR MIXED MODELS FOR CONTINUOUS IPD

In this section, we discuss different modelling options, which may be used in a one‐stage meta‐analysis using individual patient data. The data we consider have the following format: There are two treatments or exposure groups *j* = (0,1), compared in *m* studies. We will refer to the groups as the treatment group (*j* = 1) and the control group (*j* = 0). Let *Y*_*ijk*_ denote the outcome (iron level, folate level) of patient *k* in study *i* receiving treatment *j* and *X*_*ijk*_ a dummy variable to indicate the group; *X*_*ijk*_ = 0 when patient *k* in group *i* of study *i* receives the control; and *X*_*ijk*_ = 1 if the patient receives the treatment. Note that we do not introduce any covariates in this work and hence no covariate notation. In addition, no adjustment for baseline values (ANCOVA) is possible under the discussed models.

Several papers have discussed the modelling of continuous IPD in the meta‐analytic framework Higgins et al.^24^ In this paper, we present different modelling options for treatment/exposure and study effects and various ways to model the within‐study residual variance. We use the linear mixed modelling (LMM) framework^25^ and explore three modelling options of increasing complexity using LMM notation. In general, we denote fixed effects using characters from the greek alphabet and latin characters for the random effects.

### 3.1 Study‐specific fixed intercepts and fixed treatment effect model

A simple fixed treatment effect meta‐analysis model may be written as follows:

(1) $$Y_{ijk}=\beta_{0i}+\beta_{1}X_{ijk}+\epsilon_{ijk},$$

where *β*_0*i*_ is the fixed study‐specific mean of the control treatment in study *i* and *β*_1_ the mean difference between the treatment and control group across studies. For each study, a separate intercept is estimated to account for the difference in response between studies. This model assumes that the difference between the groups is the same across all studies; this is often a very unrealistic assumption. The within‐study residuals *ϵ*_*ijk*_ are assumed to follow a normal distribution. There are different options for the choice of variance of *ϵ*_*ijk*_, which we discuss later on.

### 3.2 Study‐specific fixed intercepts and random treatment effects

We relax the assumptions of the fixed treatment effect model by allowing the treatment differences to vary across studies which results in a mixed effects model where treatment differences are treated as random:

(2) $$Y_{ijk}=\beta_{0i}+\beta_{1}X_{ijk}+b_{1i}X_{ijk}+\epsilon_{ijk},$$

where *β*_1_ is the mean treatment difference and *b*_1*i*_ are the trial‐specific additional treatment differences following a normal distribution with mean equal to 0 and variance equal to $\tau_{1}^{2}$. This model estimates *m* fixed effects parameters (study intercepts) and an additional fixed effect for the mean group differences among studies. In addition, the between‐study variance is estimated, which quantifies the heterogeneity of the treatment effects across studies. The within‐study residual variances $\sigma_{\epsilon ,ij}^{2}$ are also estimated.

### 3.3 Random intercepts and treatment effects

Instead of estimating a fixed study effect per study, we can assume that the study effects are also random resulting in the following model:

(3) $$Y_{ijk}=\beta_{0}+\beta_{1}X_{ijk}+b_{0i}+b_{1i}X_{ijk}+\epsilon_{ijk},$$

where *β*_0_ is the fixed overall mean intercept, *b*_0*i*_ is the random study intercept, *β*_1_ is the mean treatment difference, and the *b*_1*i*_ is the study‐specific additional treatment differences with

$$\left[\begin{array}{c} b_{0i}\\ b_{1i} \end{array}\right]=MVN\left(\left[\begin{array}{c} 0\\ 0 \end{array}\right],\left[\begin{array}{cc} \tau_{0}^{2} & \tau_{01}\\ \tau_{01} & \tau_{1}^{2} \end{array}\right]\right).$$

The model assumes that the included studies are a random sample from a wider population of studies, an approach which is followed in analyzing multicenter trials by treating the center effects as random. There is debate about whether this is a good option.^26^ The number of estimated parameters is smaller, which could lead to more precise estimates. On the other hand, additional modelling assumptions are made. One could assume that the study‐specific treatment group differences are independent from the intercepts,^22^ but often it is more realistic to allow correlation.

### 3.4 Within‐study residual variance

The one‐stage approach allows for more flexible options when modelling the within‐study residual variance than in a two‐stage analysis where the observed variance of the difference is used.

For each of the modelling approaches in Sections 3.1, 3.2, 3.3, we explore four structures for the within‐study residual variances:

- All variances assumed different (arm‐specific and study‐specific): $\epsilon_{ijk}∼N(0,\sigma_{\epsilon ,ij}^{2})$, estimating 2 × *m* parameters. This is the most flexible approach.
- Study‐specific variances: $\sigma_{\epsilon ,ij}^{2}=\sigma_{\epsilon ,i.}^{2}$, which are equal for treated and controls, estimating *m* parameters. This may be used when study populations may differ; however, variation of outcomes in the two treatment groups is expected to be the same.
- One variance for control and one variance for treated group $\sigma_{\epsilon ,ij}^{2}=\sigma_{\epsilon ,.j}^{2}$, which are assumed to be the same for all studies, estimating two parameters. This may be used when the variation of outcomes is expected to differ between the treatment and control groups. This may be the case if treatment is compared with placebo where a larger variance in the treated group may be expected due to responders and nonresponders to the treatment.
- One overall variance: $\sigma_{\epsilon ,ij}^{2}=\sigma_{\epsilon ,..}^{2}$, estimating one variance parameter.

## 4 RECONSTRUCTION OF INDIVIDUAL PATIENT DATA FROM AGGREGATE DATA

This section describes a framework for the construction of the pseudo individual participant/patient data. The idea is as follows: If the true IPD would be available, the LMM would be used for the statistical analysis, which is likelihood based. The likelihood of an LMM depends on the data only through the observed group means and standard deviations, because they are the sufficient statistics for the parameters in the LMM. This implies that any dataset in each study with the same observed group means and standard deviations yields exactly the same maximum likelihood estimates. Our pseudo IPD method is based on these properties of sufficient statistics. We use the aggregate data to generate individual data with exactly the same observed means and standard deviations as in the original IPD data. If such a data set is analyzed with an LMM, the results will be identical to those stemming from the true IPD.

A simple algorithm to construct an appropriate data set of pseudo IPD is as follows. Let ${\bar{Y}}_{ij}$, *s* *d*_*ij*_, *n*_*ij*_ be the observed mean, standard deviation from group *j* in trial *i*. We need to generate pseudo IPD, *Y*_*ijk*_, where in each group and study, the mean is exactly equal to ${\bar{Y}}_{ij}$ and standard deviation equal to *s* *d*_*ij*_. For each group in each trial, execute the following algorithm that can be easily done in any statistical package.

1. Simulate a sample $Y_{ijk}^{*}(k=1,\text{⋯},n_{ij})$ from a certain distribution, for a example a standard normal distribution.
2. Calculate the mean ${\bar{Y}}_{ij}^{*}$ and standard deviation $sd_{ij}^{*}$ per study and arm of this sample.
3. Generate the pseudo outcomes as $Y_{ijk}={\bar{Y}}_{ij}+sd_{ij}\left(\frac{Y_{ijk}^{*}-{\bar{Y}}_{ij}^{*}}{sd_{ij}^{*}}\right)$; we obtain a sample with mean exactly equal to ${\bar{Y}}_{ij}$ and sd equal to *s* *d*_*ij*_.

Note that the sampling from the normal distribution is not essential; in fact, any arbitrary values $Y_{ijk}^{*}$ will do. For instance, one could alternatively make the first observation equal to 1 $\left(Y_{ij1}^{*}=1\right)$ and the remaining equal to 0, $Y_{ijk}^{*}=0$ for *k* = 2,…,*n*_*ij*_. The pseudo IPD in this case would be $Y_{ij1}={\bar{Y}}_{ij}+\frac{n-1}{\sqrt{n}}sd_{ij}$ and $Y_{ijk}={\bar{Y}}_{ij}-\frac{1}{\sqrt{n}}sd_{ij}$ for *k* = 2,…,*n*_*ij*_. Again, leading to a sample with mean equal to ${\bar{Y}}_{ij}$ and standard deviation equal to *s* *d*_*ij*_.

In Appendix A, we show how this algorithm can be carried out in SAS, R, and SPSS.

## 5 APPLICATION OF THE METHODS TO THE EMPIRICAL DATA

We generated pseudo IPD for the two illustrating examples and fitted the models discussed in Section 3. In LMM programs, one can choose between maximum likelihood (ML) estimation and restricted ML. The latter removes the downward bias in the variance parameters and leads to more accurate standard errors of the estimates of the parameters of the fixed part of the model. We present 12 possible model combinations stemming from the three modelling options (fixed study‐specific intercepts and fixed treatment effect model, fixed study‐specific intercepts and random treatment group effects, and both study and treatment/exposure effects treated as random) and four options to model the within‐study residual variance. We initially fitted all models using the LMM program of SAS, PROC MIXED, given that SAS has explicit options for modelling the residual variance, yet we replicated the analyses in other statistical software, R, via the nlme package^27^ and SPSS. In Appendix A, we provide details. Note that the confidence intervals of the estimates derived from different software can be different due to different method of calculating the degrees of freedom; the point estimates and their standard errors are identical.

For some of the more complex models, nonconvergence issues and boundary estimates of zero for the between‐study variance occurred. This appeared to be program‐dependent; a model could show nonconvergence in SAS but convergence in R and the other way around. The remedy was to provide more informative starting values.^28^ To compare nested models with the same fixed part but different random parts, the REML likelihood ratio test was used. To compare non‐nested models with the same fixed part, we calculated the Akaike information criterion (AIC)^29^ using the complete likelihood of the model. As a rule of thumb, an AIC difference between two models of less than two provides little evidence for one over the other; the models are considered interchangeable.^30^

In general, when we compare the LMMs fitted on the pseudo IPD with the standard DL and REML results based on the aggregate data, we notice that the estimates and standard errors of the overall effect are similar. However, the confidence interval stemming from our approach is much wider and the *P* value larger. This is due to the fact that the LMM approach does not consider the residual standard deviation per study as fixed. Using maximum likelihood takes into account that study means and residual standard deviations are estimates, resulting in larger standard errors for the group differences. However, whether the uncertainty of the estimates is effectively accounted for depends on which likelihood‐based inference method is used. In contrast to the standard Wald method, the likelihood ratio (LR) method and the score method take the uncertainty in the estimation of the variance parameters into account for significance testing and CIs. Furthermore, confidence intervals calculated in LMM are based on *t*‐distributions, to account for small number of studies. There are several methods to determine the number of degrees of freedom of the *t*‐distribution that is used to calculate the confidence interval and the *P* value. We used the default method of SAS, the containment method. SAS PROC MIXED offers four alternative methods, which produced very similar results in these examples. The Hartung‐Knapp correction^7^ applied to REML method for aggregate data has the same aim as the degrees of freedom adjustment in the LMM. However, it does not take into account the uncertainty in the estimates of the residual variance(s). Application of the Hartung‐Knapp correction leads to results very similar to the results of our final best fitted models.

### 5.1 Results on iron blood levels

We compare the results of the 12 models fitted with the pseudo IPD data to the three commonly used two‐stage meta analysis approaches based on aggregate mean differences and variances per study. The results of the iron pseudo IPD dataset are presented in Table 3. Within each of the three blocks, we present the results of linear mixed models of the same fixed part and different models for the residual variance. For comparison, the fourth block gives the results of the two‐stage analyses on the aggregate dataset.

**Table 3:** Results of iron pseudo IPD

Model		Estimate	SE	95% CI	AIC REML	−2RES LogLik	#FE	#RE	AIC ML	−2LogLik	$\tau_{1}^{2}$
Fixed study‐specific intercepts and
fixed treatment effect	$\sigma_{\epsilon ,ij}^{2}$	−6.91	2.85	(−12.5 to −1.3)	6697.7	6677.7	6	10	6734.3	6702.3	‐
	$\sigma_{\epsilon ,i.}^{2}$	−6.95	3.16	(−13.1 to −0.7)	6701.5	6691.5	6	5	6738.7	6716.7	‐
	$\sigma_{\epsilon ,.j}^{2}$	−5.83	3.13	(−11.9 to 0.3)	6699.9	6695.9	6	2	6736.8	6720.8	‐
	$\sigma_{\epsilon ,..}^{2}$	−5.82	3.15	(−12.0 to 0.4)	6698.0	6696.0	6	1	6734.9	6720.9	‐
Fixed study‐specific
intercepts and random
treatment effects	$\sigma_{\epsilon ,ij}^{2}$	−5.59	4.41	(−17.8 to 6.6)	6699.1	6677.1	6	11	6736.3	6702.3	45.1
	$\sigma_{\epsilon ,i.}^{2}$	−5.51	4.64	(−18.4 to 7.3)	6702.7	6690.7	6	6	6740.7	6716.7	51.0
	$\sigma_{\epsilon ,.j}^{2}$	−4.85	4.86	(−18.3 to 8.6)	6700.5	6694.5	6	3	6738.8	6720.9	64.3
	$\sigma_{\epsilon ,..}^{2}$	−4.86	4.86	(−18.3 to 8.6)	6698.6	6694.6	6	2	6735.0	6721.0	63.6
Random intercept and
random treatment effect	$\sigma_{\epsilon ,ij}^{2}$	−4.54	4.33	(−16.5 to 7.4)	6739.8	6713.8	2	13	6754.3	6724.3	44.2
	$\sigma_{\epsilon ,i.}^{2}$	−4.46	4.55	(−17.1 to 8.1)	6743.3	6727.3	2	8	6758.0	6738.0	49.0
	$\sigma_{\epsilon ,.j}^{2}$	−4.05	4.70	(−17.1 to 9.0)	6741.1	6731.0	2	5	6755.8	6741.8	59.0
	$\sigma_{\epsilon ,..}^{2}$	−4.06	4.71	(−17.1 to 9.0)	6739.1	6731.1	2	4	6753.9	6741.9	58.3
Aggregate RE DL		−5.57	4.38	(−14.1 to 3.0)	‐	‐	‐	‐	‐	‐	43.9
Aggregate RE REML		−5.52	4.47	(−14.3 to 3.2)	‐	‐	‐	‐	‐	‐	47.3
Aggregate RE REML
Hartung‐Knapp		−5.52	4.70	(−18.6 to 7.5)	‐	‐	‐	‐	‐	‐	47.3

Abbreviations: AIC, Akaike information criterion (smaller is better); CI, confidence interval; *#*FE, number of fixed effect parameters; LogLik, log likelihood; ML, maximum likelihood; *#*RE, number of (co)variance parameters; RES, restricted; SE, standard error; $\sigma_{\epsilon ,ij}^{2}$, study‐ and arm‐specific variances; $\sigma_{\epsilon ,i}^{2}$, study‐specific variances; $\sigma_{\epsilon ,j}^{2}$, two variance parameters; one for control and one for treatment; $\sigma_{\epsilon ,..}^{2}$, one overall variance; $\tau_{1}^{2}$, between‐study variance.

Within each block, the model with all within‐study residual variances assumed to be free was compared with the more restricted within‐study structures. For instance, for the fixed study‐specific intercept and fixed treatment effect model assuming $\sigma_{\epsilon ,ij}^{2}$ versus the study‐specific variances $\left(\sigma_{\epsilon ,i.}^{2}\right)$ model, the test statistic (−2 restricted log likelihood value) was found to be equal to 6691.5 − 6677.7 = 13.85. The test statistic follows a *χ* ^2^ distribution with five degrees of freedom, the difference of the random parameters under the two models, giving a *P* value of 0.018. In all blocks, the full model fitted significantly better. Therefore, we can conclude that the variation of iron values differs between studies and differs between patients and controls.

We compare these models in the different blocks (fixed, fixed‐random, and random‐random) with the AIC ML criterion. The AIC values within the blocks are very similar, which suggest that one may adopt one of the simpler models if opting for a parsimonious model. The lowest value, 6734.3, was found for the model assuming fixed study‐specific intercepts and a fixed treatment effect, with the model with fixed study‐specific intercepts and random treatment effects as a close competitor. We choose a random effects model here, because the estimated values of *τ* were quite substantial, ranging from 6.6 to 8 μg/dL, which was larger than the estimated mean difference between the two groups. This suggests substantial heterogeneity of the effects. We thereby follow the generally accepted arguments in favor of random treatment effects meta‐analysis.^1^, ^2^ The mean difference between the Alzheimer disease group and the healthy control group was equal to −5.59 μg/dL with 95*%* interval of −17.8 to 6.6 μg/dL.

### 5.2 Results on folate plasma levels

The results of the folate pseudo IPD dataset are presented in Table 4. Here, the models assuming a more flexible within‐study residual variance structure yielded larger point estimates of the mean difference compared with the models assuming the same variance across studies (first two rows of each block).

**Table 4:** Results of folate pseudo IPD

Model		Estimate	SE	95% CI	AIC REML	−2RES LogLik	*#*FE	*#*RE	AIC ML	−2LogLik	$\tau_{1}^{2}$
Fixed study‐specific intercepts and
fixed treatment effect text	$\sigma_{\epsilon ,ij}^{2}$	−3.24	0.15	(−3.5 to −2.9)	31 767.9	31 643.9	32	62	31 851.5	31 663.5	‐
	$\sigma_{\epsilon ,i.}^{2}$	−3.29	0.13	(−3.5 to −3.0)	31 963.9	31 901.9	32	31	32 053.3	31 927.3	‐
	$\sigma_{\epsilon ,.j}^{2}$	−2.98	0.36	(−3.7 to −2.2)	35 181.2	35 177.2	32	2	35 307.1	35 239.1	‐
	$\sigma_{\epsilon ,..}^{2}$	−3.13	0.38	(−3.8 to −2.4)	35 374.5	35 372.5	32	1	35 504.3	35 438.2	‐
Fixed study‐specific
intercepts and random
treatment effects	$\sigma_{\epsilon ,ij}^{2}$	−3.87	0.63	(−5.1 to −2.6)	31 636.1	31 510.1	32	63	31 748.5	31 558.5	9.81
	$\sigma_{\epsilon ,i.}^{2}$	−3.91	0.63	(−5.2 to −2.6)	31 810.5	31 746.5	32	32	31 920.8	31 792.8	9.97
	$\sigma_{\epsilon ,.j}^{2}$	−3.68	0.63	(−4.9 to −2.4)	35 164.0	35 158.0	32	3	35 308.2	35 238.2	6.41
	$\sigma_{\epsilon ,..}^{2}$	−3.71	0.62	(−4.9 to −2.4)	35 358.6	35 354.6	32	2	35 501.4	35 433.4	6.31
Random intercept and
random treatment effect	$\sigma_{\epsilon ,ij}^{2}$	−3.98	0.64	(−5.2 to −2.6)	31 841.3	31 711.3	2	65	31 848.3	31 714.3	9.97
	$\sigma_{\epsilon ,i.}^{2}$	−4.03	0.64	(−5.3 to −2.7)	32 015.9	31 947.9	2	34	32 022.9	31 950.9	10.22
	$\sigma_{\epsilon ,.j}^{2}$	−3.65	0.63	(−4.9 to −2.3)	35 369.8	35 359.8	2	5	35 376.8	35 362.8	6.71
	$\sigma_{\epsilon ,..}^{2}$	−3.67	0.63	(−4.9 to −2.3)	35 564.7	35 556.7	2	4	35 571.8	35 559.8	6.74
Aggregate RE DL		−3.80	0.48	(−4.7 to −2.8)	‐	‐	‐	‐	‐	‐	5.17
Aggregate RE REML		−3.88	0.63	(−5.1 to −2.6)	‐	‐	‐	‐	‐	‐	9.88
Aggregate RE REML
Hartung‐Knapp		−3.88	0.63	(−5.1 to −2.6)	‐	‐	‐	‐	‐	‐	9.88

Abbreviations: AIC, Akaike information criterion (smaller is better); CI, confidence interval; *#*FE, number of fixed effect parameters; LogLik, log likelihood; ML, maximum likelihood; *#*RE, number of (co)variance parameters; RES, restricted; SE, standard error; $\sigma_{\epsilon ,ij}^{2}$, study‐ and arm‐specific variances; $\sigma_{\epsilon ,i}^{2}$, study‐specific variances; $\sigma_{\epsilon ,j}^{2}$, two variance parameters, one for control and one for treatment; $\sigma_{\epsilon ,..}^{2}$, one overall variance; $\tau_{1}^{2}$, between‐study variance.

Again, the model with all variances free $\left(\sigma_{\epsilon ,ij}^{2}\right)$ fitted significantly better than the simpler models. The models assuming random treatment effects gave very similar results with statistically significant lower levels for the disease group compared with the healthy elderly controls. The AIC ML value was smallest for the fixed study‐specific intercepts and random treatment effects with all within‐study residual variances assumed to be free; hence, we adopt this as the final model. The mean difference in folate plasma levels between Alzheimer disease patients and healthy cognitive controls was found equal to −3.87 nmol/L with a 95*%* interval ranging from −5.1 to −2.6 nmol/L. The estimates derived from the second block of models allowing for more flexible structure of the within‐study residual variance were almost identical to the estimates stemming from the aggregate data analysis using REML estimation and REML with the HK correction method due to the much larger number of studies than in the iron levels example. The DL method yielded much smaller standard errors.

## 6 SIMULATION STUDY

We performed a simulation study in SAS to study the observed performance of the pseudo IPD method. In both datasets, the confidence intervals of the more flexible IPD models were larger than the DL and REML methods and very similar to the REML method with Hartung‐Knapp correction.^7^ To check these findings, we designed a simulation study ranging the number of studies and the number of subjects per arm under the most complex model structure that we introduced in this paper, random intercepts and treatment effects model (Section 3.3), with one within‐study variance for control and one within‐study variance for the treated group (arm‐specific). Legha et al^31^ recently performed an extensive simulation study comparing the study‐specific fixed intercepts and random treatment effects model with the random intercepts and random treatment effects model under various scenarios while ranging the estimation method (ML and REML) and the CI derivation approaches. They concluded that there were generally no differences between the two competing models in terms of mean bias, empirical SE, or MSE under any simulation scenario.

In the simulation, we compare the coverage probability at 95% nominal value, taking the proportion of the number of times the estimated 95% confidence interval included the true value of *θ*, the bias, and the mean squared error (MSE).

### 6.1 Data generation

The simulation study generated IPD from the random intercepts and treatment effects model (Section 3.3), with one within‐study variance for control and one within‐study variance for the treated group (arm‐specific). We varied the following parameters:

- Number of studies =6,12
- Number of subjects per arm =5,10,40
- Residual standard deviation in treated group =4
- Residual standard deviation in control group =1
- Mean treatment effect; *θ* = 3
- Diagonal variance‐covariance structure for the random study intercept and study‐specific treatment differences
  $$M=\left[\begin{array}{cc} \tau_{0}^{2} & \tau_{01}\\ \tau_{01} & \tau_{1}^{2} \end{array}\right]=\left[\begin{array}{cc} 4 & 0\\ 0 & 2 \end{array}\right].$$

We performed 500 simulations per combination (number of studies, number of subjects per arm). Per simulated dataset, aggregate summary data were calculated by arm and study (means, standard deviations, and number of observations). Then these aggregate summary data were used to generate pseudo IPD. The original individual patient data and the pseudo IPD were analyzed using PROC MIXED. Here, estimation was performed using REML with the containment method to calculate confidence intervals. The aggregate data were analyzed using the DL approach and REML method. For the latter, the 95% confidence interval was constructed using a *t*‐distribution with *k* − 1 degrees of freedom, where *k* corresponds to the number of studies.

### 6.2 Result

The results of the coverage probabilities, mean bias, and MSE are summarized in Table 5. Analysis of the true data and the pseudo IPD yielded exactly the same results in each simulation run. In a few runs of the small number of studies and small number of subjects per arm scenario, we encountered convergence issues of the true IPD due to between‐study correlation estimated equal to 1.

**Table 5:** Simulation study results[Link]

	(*n*_0_,*n*_1_)	**i = 6** Studies			**i = 12** Studies		
		Pseudo IPD	REML HK	DL	Pseudo IPD	REML HK	DL
Coverage probability	(5, 5)	96.2%	95.6%	89.4%	95.4%	95.2%	90.4%
	(10, 10)	96.0%	95.6%	92.6%	95.4%	95.0%	92.4%
	(40, 40)	94.8%	94.8%	87.8%	94.8%	94.6%	92.6%
Bias	(5, 5)	0.0583	0.0508	0.0159	−0.0058	−0.0061	−0.0040
	(10, 10)	0.0297	0.0300	0.0300	−0.0014	−0.0015	−0.0010
	(40, 40)	−0.0139	−0.0139	−0.0141	−0.0103	−0.0104	−0.0117
Mean squared error (MSE)	(5, 5)	0.8581	0.8603	0.9958	0.4653	0.4668	0.5621
	(10, 10)	0.6052	0.6050	0.6514	0.3062	0.3068	0.3521
	(40, 40)	0.4050	0.4046	0.4034	0.2000	0.1998	0.1989

Abbreviations: DL, DerSimonian‐Laird; *n*_0_, number of subjects in control group; *n*_1_, number of subjects in exposure group; REML HK, restricted maximum likelihood with Hartung‐Knapp correction. Coverage of the calculated 95% confidence intervals, mean bias, and MSE obtained with the different methods.

Overall, the pseudo IPD approach showed coverage probabilities very close to 95%. The coverage probability for estimates derived from the DL method was found to be always below the nominal level indicating that the confidence intervals are too small. To facilitate comparisons with the REML Hartung‐Knapp correction, we imported the SAS results into R and fitted the aforementioned model. The resulting coverage probabilities under REML HK were very similar to the pseudo IPD. The bias was small for all estimates. The pseudo IPD approach showed almost identical MSE values compared with the REML HK corrections across the various scenarios. The MSE of the DL was larger when the study sizes were small.

## 7 DISCUSSION

We have provided a general framework to generate pseudo individual patient data from aggregate meta‐analysis data with continuous outcomes. Any likelihood‐based analysis of the pseudo IPD leads to identical results as the unknown true IPD. The pseudo IPD can be analyzed in standard statistical software by standard statistical methods. This brings the meta‐analysis of continuous outcome data back into mainstream statistics. All analyses can be done using the linear mixed model (LMM), a standard statistical tool nowadays and available in all general statistical packages. Thereby meta‐analysis can profit from all the statistical methods, tools and software that is developed for the LMM. The LMM is a very general and flexible model, which gives the meta‐analyst a lot of freedom and possibilities. We mention a number of advantages of this approach. Of course, a first, practical, advantage is that the meta‐analyst can stick to his/her favorite statistical software, without the need to call upon other special purpose meta‐analysis programs.

In this article, we have focused on the most simple meta‐analysis situation of comparing two treatment or exposure groups without covariates. However, since the LMM is a very general framework, extensions to more complex situations are rather straightforward. Extension to meta‐regression is possible by adding study‐ or group‐level covariates to the model; however, we do not address the methodology for including covariates or treatment‐covariate interactions in this work. Extension to comparing three or more treatment or exposure groups is straightforward, by extending the fixed part of the model with additional treatment group indicators and the random part of the model with extra random treatment effects. Since the LMM allows missing groups, this is also the direct extension to network meta‐analysis. For network meta‐analysis, the LMM offers a big variety of models for the variances and correlations of the random treatment effects, many more than those available in special purpose network meta‐analysis software.

In addition, the LMM approach offers three general methods to test the overall treatment effect and to construct confidence intervals: Wald method, the likelihood ratio method, and the score method, albeit the last seems to be very rarely used and is not implemented in most LMM software. The most routinely used is the Wald‐type method, which assumes a normal distribution to calculate the *P* value and the confidence interval and thereby does not account for the uncertainty in the estimates of the between‐studies and within‐study residual variances. The method has often shown coverage probability below the nominal 0.95 level due to the normality assumption and the large‐sample approximation (in terms of number of studies) assumption. Therefore in all LMM software, a degrees of freedom adjustment is implemented, which replaces the standard normal distribution by a *t*‐distribution with a certain number of degrees of freedom. There are several methods to calculate the number of degrees of freedom. Most LMM software, for instance, SPSS, uses only one method, but other programs, for instance, SAS, offers the user a choice of up to five different methods. It is a matter of further research to investigate what is the best method for the typical meta‐analysis data structures. Notice that the degrees of freedom adjustment in the LMM has the same aim as the Hartung‐Knapp correction in an aggregate data meta‐analysis. Therefore, this correction is not anymore needed in the LMM approach. An alternative for Wald method is the likelihood ratio method.^32^ This method automatically takes into account the uncertainty in the estimates of the variance components, including the within‐study residual variance(s), and a degrees of freedom correction is not needed. Likelihood ratio tests are easily performed in any LMM software, and the corresponding confidence intervals are easily calculated. The likelihood ratio method also offers the possibility to test more complex null hypotheses where one might be interested in, for instance, the composite null hypothesis that the mean and the variance of the treatment effect are both zero. In some meta‐analysis applications, this is a more appropriate null hypothesis than the usual one.^33^

The LMM also provides an easy way to perform control rate regression, eg, previous studies.^34^, ^35^, ^36^ For instance, in our examples, this would answer the question whether the difference in plasma level of the micronutrients between Alzheimer patients and healthy controls is related to the mean level in the healthy control population. The natural model to study this relationship is the model with random study‐specific intercepts. In this model, the correlation is estimated, and it can be tested using the likelihood ratio test by comparing with a model where the covariance matrix of the random effects is assumed to be of variance components structure rather than unstructured.

A nice feature of the approach proposed in this paper is that it allows to model the within‐study residual variances. For instance, this can be utilized to study whether the variances in the outcome variable are heterogeneous across trials, which was indeed the case in our example datasets. Another interesting question might be to investigate whether the variance in the treated group is larger than in the control group. If the meta‐analysis includes double‐blind placebo‐controlled clinical trials as input data, observing significantly larger variances in the treatment group might be an interesting finding suggesting heterogenous response even if the overall treatment effect is not significant.

The LMM comes with many elaborated methods for goodness‐of‐fit and regression diagnostics. These can be very worthwhile in meta‐analysis applications as proposed in this paper. For instance, it might be very interesting to identify individual studies, which drive the results. The implemented methods make it very easy to find the studies that are most influential for the estimate of the overall treatment effect, its standard error, the *P* value, or for the estimate of the between studies variance.

A major advantage of having IPD for continuous outcomes is to adjust for baseline, using an analysis of covariance (ANCOVA) approach. However, often in practice, there is enough aggregate information given in the study reports to generalize our pseudo IPD approach to this case as well. Suppose from the study reports we can extract mean and standard deviation at baseline and at end of treatment per group, together with the correlation coefficient. The latter can often be calculated from the standard errors of the differences with baseline or a *P* value. Then it is possible to construct a pseudo IPD dataset for which the ANCOVA recovers exactly the ANCOVA on the true IPD. Also, it can be studied whether the treatment effect is modified by the baseline variable, again giving the same results if one had the true IPD. Working out the details of this is beyond the scope of this paper.

An additional advantage of our novel approach is that it provides an immediate solution to the difficulty of gaining access to the raw individual patient data. There are cases where access to data is not possible due to time and cost constrains, confidentiality of data, or unwillingness of the researchers to provide them. Our proposed method can be valuable in cases where IPD are available from some but not all studies of interest; therefore, a combination of originally available IPD and pseudo IPD derived from the aggregate data of the remaining studies could profit the analysis.

Finally, given the comparable performance of the aggregate data MA using the HK correction^7^ and our approach allowing flexible options for the modelling of the within‐study residual variances, our recommendation to the applied researchers is that either approach can be considered rather interchangeably unless specific interest lies in modelling more realistic (or potentially complex) residual variance structures across studies which can be implemented only by pseudo IPD approach.

## CONFLICT OF INTEREST

The author reported no conflict of interest.

## APPENDIX A CODE

We have analyzed the iron and folate datasets of our paper using SAS, SPSS, and R. The code for the three different programs on the iron dataset is given below. In each of the provided codes, the following family of models are fitted

1. Both study and treatment effect fixed
2. Study effect fixed and treatment effect random
3. Both treatment effect and study effect random

Within each family, four different variance structures are considered:

1. Arm‐specific variances estimated (per study and group a separate variance estimate), $\sigma_{\epsilon ,ij}^{2}$
2. Study‐specific variances estimated (per study a different variance parameter, which is the same in the two treatment groups), $\sigma_{\epsilon ,i.}^{2}$
3. Group‐specific variances estimated (one variance for all control groups; one variance for all treatment groups), $\sigma_{\epsilon ,.j}^{2}$
4. One residual variance, $\sigma_{\epsilon ,..}^{2}$
